# EHMTI-0220. Cortical excitability in episodic cluster headache

**DOI:** 10.1186/1129-2377-15-S1-E11

**Published:** 2014-09-18

**Authors:** G Cosentino, B Fierro, S Brancato, P Paladino, R Baschi, S Talamanca, S Indovino, F Brighina

**Affiliations:** 1 Department of Experimental Biomedicine and Clinical Neuroscience (BioNeC), University of Palermo, Palermo, Italy

## Background

Cluster headache (CH) is a severe primary headache disorder, whose pathophysiological processes remain largely unknown. Along with central disinhibition of the trigeminal nociceptive system and hypothalamic impairment, a cortical involvement has been supposed.

## Aim

To evaluate cortical excitability in episodic CH patients by using different paradigms of transcranial magnetic stimulation (TMS).

## Methods

Twenty-five patients with episodic CH and thirteen healthy subjects underwent an experimental session where we evaluated, in both hemispheres, motor-cortical response to: 1) single-pulse TMS: i.e. motor threshold (MT); input-output (IO) curves and cortical silent period (CSP) and 2) paired-pulse TMS: i.e. intracortical facilitation (ICF) and short intracortical inhibition (SICI). Thirteen patients were evaluated outside bout, while the remaining twelve patients were inside bout at the time of recording.

## Results

We showed increased ICF values in the hemisphere ipsilateral to the side of pain in patients evaluated both outside and inside bout. Differently, IO curves showed increased slope in both hemispheres in patients examined outside bout, but only in the hemisphere contralateral to the affected side in those evaluated during bout.

## Conclusions

Our results show a condition of increased cortical excitability in episodic CH both outside and inside bout. Interestingly, cortical excitability was greater in the hemisphere ipsilateral to the side of pain in patients outside bout, but decreased in patients inside bout possibly due to activation of compensatory inhibitory mechanisms of cortical excitability. Along with subcortical and peripheral mechanisms, changes in cortical excitability could also play an important role in the pathophysiology of CH.

No conflict of interest.

